# Trachway® flexible stylet facilitates the correct placement of double-lumen endobronchial tube: a prospective, randomized study

**DOI:** 10.1186/s12871-022-01800-8

**Published:** 2022-08-15

**Authors:** Hung-Te Hsu, Yi-Wei Kuo, Chao-Wei Ma, Miao-Pei Su, Kuang-Yi Tseng, Chin-Ling Li, Kuang-I Cheng

**Affiliations:** 1 Department of Anesthesiology, Kaohsiung Medical University Chung-Ho Memorial Hospital, No.100, Tzyou 1st Rd., Sanmin Dist., 80756 Kaohsiung City, Taiwan (R.O.C.); 2 Department of Anesthesiology, Faculty of Medicine, College of Medicine, Kaohsiung Medical University, Kaohsiung, Taiwan

**Keywords:** Thoracic surgery, Trachway flexible stylet, Double-lumen endobronchial tubes, Mucosal complication of tracheal carina

## Abstract

**Background:**

The mainstream facilitation of one-lung ventilation is using double-lumen endobronchial tubes. However, it is more difficult to be positioned properly and more likely to cause airway injuries. How to place double-lumen endobronchial tubes rapidly and correctly is important for thoracic anesthesiologists.

**Methods:**

One hundred eight patients with an American Society of Anesthesiologists physical status of I to III were 20 years of age or over, and required one-lung ventilation for thoracic surgery. They were randomly assigned to the conventional technique group (*n* = 36), the flexible fiberoptic bronchoscopy group (*n* = 36), or the Trachway® flexible stylet group (*n* = 36). The primary endpoint was the time needed for intubation. T1, the time from the tip of the blade passing between the patient’s lips to identification of the vocal cords; and T2, the time from identification of the vocal cords to the bronchial lumen was in the correct position.

**Results:**

T1 had no significant difference between groups, but T2 was significantly shorter in the Trachway® flexible stylet group (*p* < 0.0001) and longer in the conventional technique group (*p* < 0.0001).

**Conclusions:**

Using Trachway® flexible stylet for correct placement of double-lumen endobronchial tubes not only significantly shortened the intubation time, but also reduced incidence of carinal injuries. It is an alternative, and a choice with good safety.

**Trial registration:**

ClinicalTrials.gov Identifier: NCT02364622, 18/02/2015, Retrospectively registered.

**Supplementary Information:**

The online version contains supplementary material available at 10.1186/s12871-022-01800-8.

## Introduction

For thoracic surgical procedures, the anesthesiologists use lung-isolation techniques to achieve one-lung ventilation (OLV) [1]. So far, the mainstream facilitation of OLV is using double-lumen endobronchial tubes (DLTs) [2, 3]; however, due to large size and complicated structure, DLTs are more likely to cause airway injuries [4, 5]. Because of the underlying lung disease, patients undergoing thoracic surgery may be less tolerant of apnea; therefore, how to place DLTs correctly and rapidly is an important issue.

**Fig. 1:**
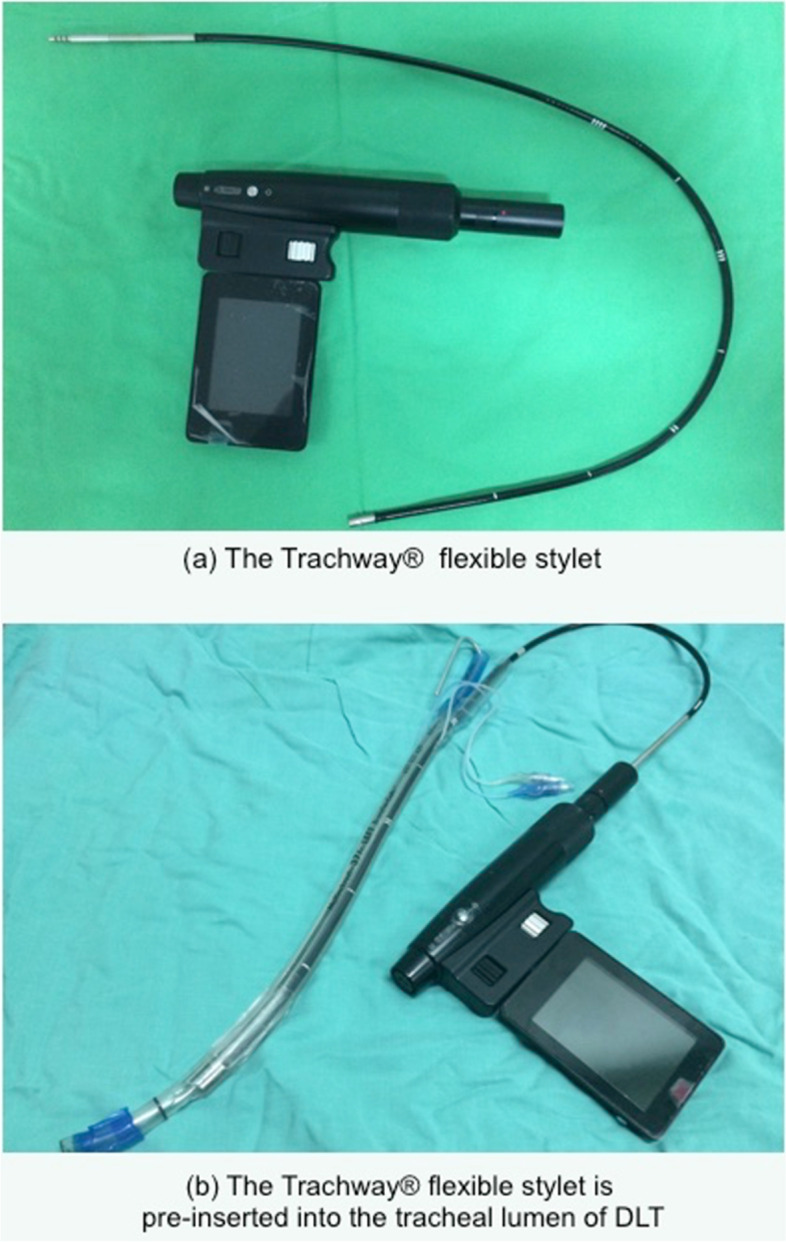
The Trachway® flexible stylet

The Trachway® flexible stylet (Trachway®; Biotronic Instrument Enterprise, Tai Chung, Taiwan) is a soft and flexible airway device (Fig. 1a) with a length of 65 cm and an external diameter of 5.0 mm. A light source and a digital camera are positioned at the distal end of the stylet. The camera has an 85° diagonal field-of-view complementary metal oxide semiconductor image sensor. The stylet is built with two white LEDs as a light source with a defogging function. The proximal end of the stylet can be plugged into the rechargeable handle. Through an adjustable 3-inch monitor attached to the handle, the video image can be observed. Clinically, it can serve as an introducer to confirm the correct position of endotracheal tube intubation, and to facilitate nasopharyngeal intubation.

Traditionally, the flexible fiberoptic bronchoscopy (FFB) is used to guide and adjust the position of DLT. Unlike FFB, the Trachway® flexible stylet can be detached from the video monitor. Therefore, the flexible stylet is allowed to pre-insert into the tracheal lumen of DLT. After intubation, we connect it back to the monitor and adjust bronchial lumen to the correct position. Our aim was to investigate the clinical performance and the efficacy of this stylet in comparison with FFB. The primary outcome was the efficacy of DLT correct positioning, with secondary outcomes being the proportion of correct positioning, the incidence of postoperative complaints, such as sore throat and hoarseness, and airway injuries around the tracheal carina.

## Material and methods

This study was conducted in accordance with the Declaration of Helsinki tenets, and approved by the Institutional Research Board of Kaohsiung Medical University Hospital (No. KMUH-IRB-20130194). Trial registration: ClinicalTrials.gov Identifier: NCT02364622, 18/02/2015, Retrospectively registered. All patients gave their informed consent and signed the appropriate form between October 2013 and December 2014. Inclusion criteria were patients 20 years of age or over, American Society of Anesthesiologists physical status I-III, undergoing elective thoracic surgery with OLV under general anesthesia. We excluded subjects with limited understanding of local language or learning difficulties, diseases with gastro-esophageal reflux, pregnancy, scheduled for surgery over six hours, tracheostomy or delayed extubation with postoperative intensive care. In addition, patients with indications of a potentially difficult intubation—including limited mouth opening (< 3 cm), limited neck extension (< 35°), a distance between chin tip to upper margin of thyroid cartilage less than 6 cm, or sternomental distance less than 12.5 cm while patients’ head was fully extended were also excluded [6]. Patients were randomly assigned into the conventional group (Group C), the flexible fiberoptic bronchoscopy group (Group F), or Trachway® flexible stylet group (Group T) by codes kept in a sealed opaque envelope that were generated by computer.

**Fig. 2:**
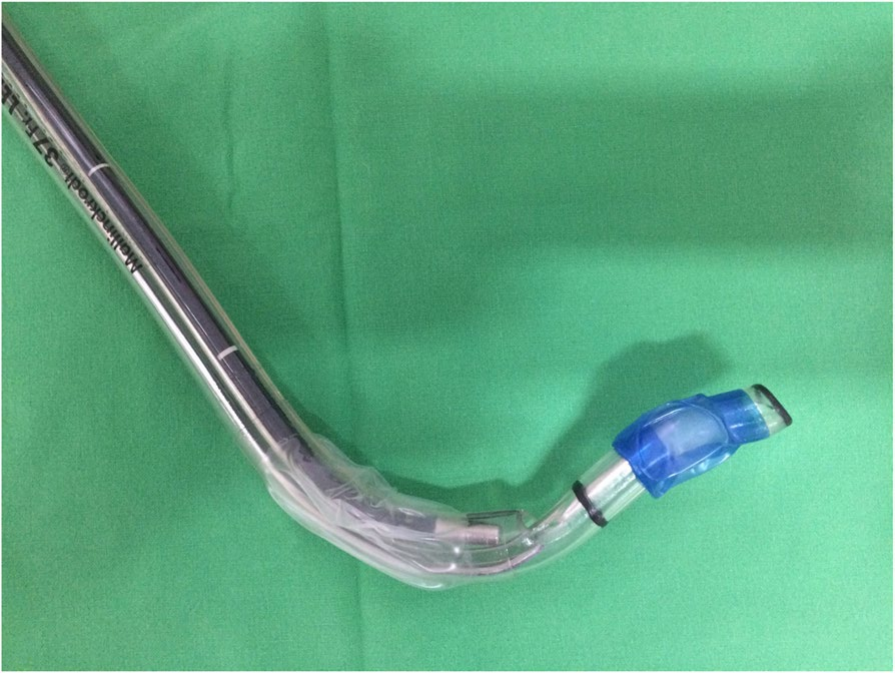
The pre-curved left-sided DLT

In the operating room, patients were monitored with electrocardiography, peripheral arterial oxygen saturation and blood pressure through radial artery cannulation on wrist. In all group, left-sided DLTs (Broncho-Cath®; Mallinckrodt, St. Louis, MO) were selected with 37 Fr for male patient and 35 Fr for female patient. We pre-curved the distal 8–10 cm of DLT approximately 90° with the DLT-malleable stylet (Mallinckrodt, Mansfield, MA, USA) through the bronchial lumen [7], and it was angled out to the right side to cover the distal orifice of the tracheal lumen (Fig. 2). The randomized process of grouping was blinded from the patients and the anesthetists who collected the postoperative data. Intravenous thiamylal (5 mg.kg^−1^), fentanyl (2 mcg.kg^−1^), and cisatracurium (0.15 ~ 0.2 mg.kg^−1^) were used for the induction of anesthesia to facilitate intubation after pre-oxygenation. Propofol was administered (1.0 mg.kg^−1^) prior to intubation to blunt intubation-related hemodynamic responses.

All bronchial and tracheal cuffs of the left-sided DLT were thinly lubricated with SURGILUBE® sterile surgical lubricant (E. FOUGERA & CO. Melville, New York, a division of Nycomed US Inc.). The intubation (shaped like a hockey stick) was performed with GlideScope® video-assisted laryngoscope in group C and F by two board certified anesthesiologists, who had performed at least 300 tracheal intubations with the GlideScope®. Once the bronchial cuff of the DLT was passed beyond the vocal cords, the metal stylet was removed. The DLT was rotated 90 degrees clockwise to make bronchial lumen toward the left side. In group C, the DLT was advanced to main bronchus until resistance was felt. The position of bronchial lumen was confirmed and adjusted by FFB. In Group F, FFB was inserted through the bronchial lumen. After identification of the tracheal carina, FFB entered the left main bronchus and served as a guide to advance the bronchial lumen. [8].

In Group T, the Trachway® flexible stylet was inserted into the tracheal lumen of DLT (Fig. 1b). The intubation was also performed with GlideScope®. After the DLT was rotated 90 degrees clockwise, the Trachway® flexible stylet was connected to the video monitor. The advance of DLT was guided by the image of monitor. When the tracheal carina was visible on the monitor, the tip of bronchial lumen of DLT was directed toward and inserted into the left main bronchus carefully. The advancement of DLT was stopped immediately when the blue cuff of bronchus lumen entered completely into the left bronchus. At last, the breathing sound of left chest would be checked by stethoscope routinely in all groups.

**Fig. 3:**
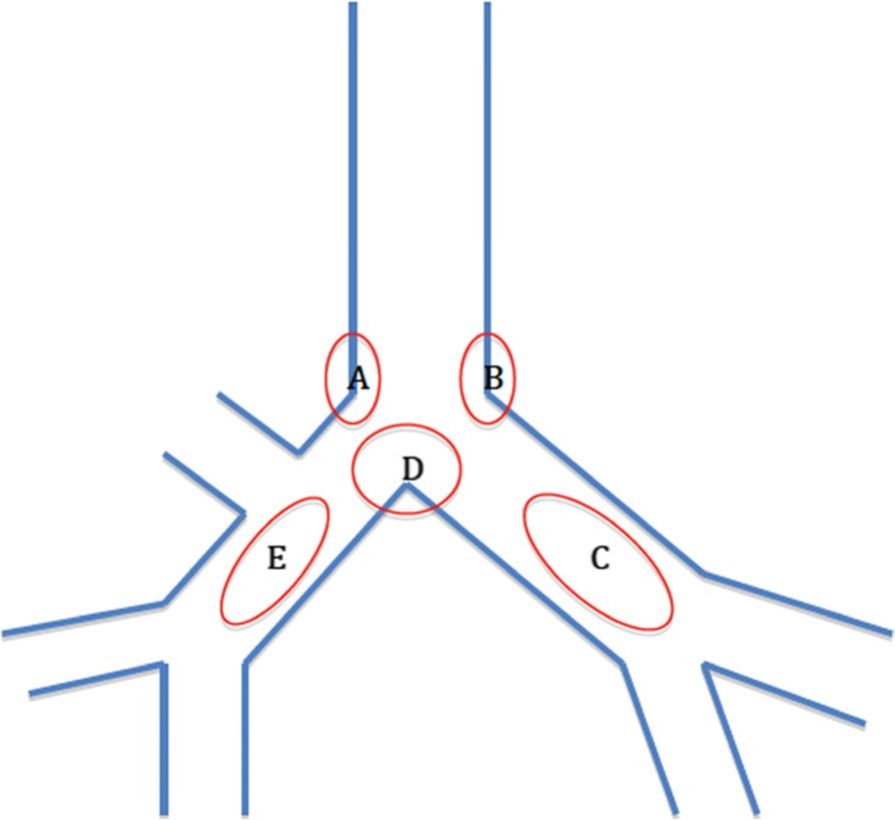
Five sub-regions of tracheal carina. **A** the entrance of right main bronchus. **B** the entrance of left main bronchus. **C** the left main bronchus. **D** the main carina. **E** the right main bronchus

Intubating data were recorded by an independent observer. The primary outcome was the time taken for DLT placement. The total time (T total) was calculated from the time when the blade tip of GlideScope® passed between the patient’ s lips to DLT was certainly in the correct position. This time was subdivided into: T1, the time from the tip of the blade passing between the patient’ s lips to identification of the vocal cords; and T2, the time from identification of the vocal cords to the bronchial lumen was in the correct position. The secondary outcomes were the success rate of first-attempt, hemodynamic responses, and complications related to DLT placement. Mean blood pressure and heart rate were recorded before induction of anesthesia (baseline), pre-intubation (pre-I) and 1, 3 and 5 min post-intubation (post-I 1, post-I 3 and post-I 5 respectively). An otolaryngologist blinded to the study examined the oral cavity, pharynx and larynx 5 min after intubation for signs of lacerations or bleeding. At the end of surgery, the patient was turned to the supine position. The DLT would be removed in regular spontaneous breathing with tidal volumes exceeded 5 ml.kg^−1^ [9]. An investigator, blinded to the group assignment, inserted FFB to search for main carina or bronchial injuries. We divided the tracheal carina into five sub regions: A: the entrance of right main bronchus, B: the entrance of left main bronchus, C: the left main bronchus, D: the main carina, and E: the right main bronchus (Fig. 3). The extent of hoarseness, sore throat and odynophagia were recorded by another independent anesthesiologist blinded to the study on the first four postoperative days. Participants scored sore throat and odynophagia on a visual analog scale from 0, indicating ‘none’ to 10: scores above 0 were subsequently categorized as mild (1– 3), moderate (4– 6) or severe (7– 10). Hoarseness was classified as absent (0), subjective (1), observed by the anesthesiologist (2) or aphonic (3).

According to our pilot data, the mean difference in time for intubation was 9.2 s (20% mean difference) with a standard deviation of 11 s. A priori power analysis revealed that 31 participants were needed in each group to detect a difference with a power of 0.9 at an α level of 0.05. Before the between-groups comparisons, the Kolmogorov–Smirnov test was used to test the distribution normality of data. Student’s *t*-test, the chi-square test and Fisher’s exact test were used to compare the groups. In addition, hemodynamic data such as heart rate and blood pressure were analyzed with repeated-measure analysis of variance (ANOVA) for intra- and intergroup comparisons. Bonferroni’s post hoc tests were undertaken where appropriate. Data are presented as the mean ± standard deviation, or the number and proportion as appropriate. SPSS 17.0 software (Apache Software Foundation, Forest Hill, MD, USA) was used for all statistical analyses.

## Results

**Fig. 4:**
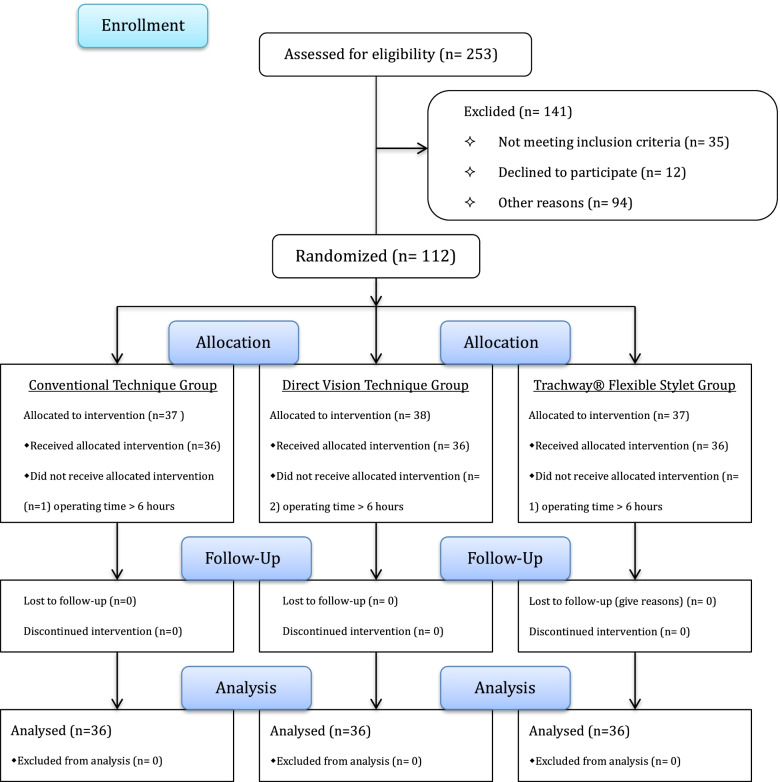
Flow Diagram of Trachway® flexible stylet facilitates the accurate placement of double-lumen endobronchial tube

Two hundred and fifty-three patients undergoing thoracic surgery were approached to participate in the study, but 141 did not meet the inclusion criteria. Ultimately, 112 patients were enrolled, but four were excluded. The data of 108 patients were subject to final analysis (Fig. 4).

The clinical and demographic characteristics, upper airway characteristics as well as anesthesia duration of each group were broadly comparable (Table 1). There was no significant difference between groups. Time required to complete DLT placement**,** and number of positioning attempts in all groups are shown in Table 2. The mean total DLT placement time was significantly shortest in Group T and longest in Group C (69.2 ± 11.7 vs 146.8 ± 31.0 s, 95% confidence interval 77.6 (66.587 to 88.613), *p* < 0.001). T1 between all groups had no significant difference, but T2 was significantly shorter in Group T and longer in group C (47.9 ± 11.2 vs 122.1 ± 28.7 s, 95% confidence interval 74.3 (64.059 to 84.541), *p* < 0.001). The first-attempt success rate for the correct placement of bronchial lumen was lowest in Group C (25%, *p* < 0.001), but no statistic difference was revealed between groups T and F. Peripheral oxygen saturation was maintained at 99 – 100% in all patients during DLT placement.

**Table 1:** Patient demographic characteristics, airway characteristics and duration of anesthesia. Data are expressed as mean ± SD or frequency where applicable

	**Trachway® Group (T, *n* = 36)**	**Conventional Group (C, *n* = 36)**	**Fiberscope Group (F, *n* = 36)**	***P* value**
Gender Male: Female	21: 15	23:13	15:21	0.143
Age (yrs)	46.2 ± 15.3	49.6 ± 14.2	47.0 ± 12.8	0.565
Body Weight (kg)	63.2 ± 13.7	60.3 ± 8.8	64.5 ± 13.0	0.319
Height (cm)	163.1 ± 10.2	161.7 ± 8.2	163.4 ± 9.5	0.732
BMI	23.7 ± 4.1	23.2 ± 3.9	24.1 ± 4.2	0.699
ASA class I/II/III	1/21/14	1/18/17	1/21/14	0.951
Mouth Open (cm)				
Active	4.4 ± 0.8	4.5 ± 0.8	4.6 ± 0.8	0.956
Passive	3.8 ± 0.7	3.8 ± 0.5	4.0 ± 0.4	0.339
TMD (cm)	8.6 ± 1.1	8.8 ± 1.6	8.8 ± 1.2	0.603
Mallampati Class I/II/III/IV	12/17/7/0	15/13/8/0	12/16/8/0	0.829
CL Grading I/II/III/IV	25/9/2/0	24/12/0/0	29/7/0/0	0.207
Anesthesia time (min)	183.5 ± 63.4	180.6 ± 73.5	173.9 ± 52.3	0.807

The anesthesia time: from induction of anesthesia to extubation at end of the surgery

*TMD* Thyromental distance, *CL grading* Cormack-Lehane grading

**Table 2:** Time required to complete DLT placement**,** and number of positioning attempts. Data are expressed as mean ± SD or frequency where applicable

	**Trachway® Group** **(T, *n* = 36)**	**Conventional Group** **(C, *n* = 36)**	***P* value** **(C VS T)**	**Fiberscope Group** **(F, *n* = 36)**	***P* value** **(F VS T)**
**Time interval** (**seconds)**					
**T1**	21.9 ± 5.1	24.2 ± 6.8	0.105	22.6 ± 3.8	0.459
**T2**	47.8 ± 11.2	122.1 ± 28.7	< 0.001	72.0 ± 22.1	< 0.001
**T Total**	69.2 ± 11.69	146.8 ± 31.0	< 0.001	95.5 ± 24.6	< 0.001
**Attempts:** **1/2/3**	32/3/1	9/25/2*^¥^	< 0.001	33/3/0	1
**First-Attempt success rates**	88.89% (32/36)	25% (9/36)	< 0.001	91.67% (33/36)	1

Attempts: 1 = 1, 2 = 2, 3 ≥ 3

The hemodynamic responses to intubation were significantly different between groups. The changes of heart rate were comparable between groups C and F, but decreased in Group T at 3 min after intubation (*p* < 0.001). The changes of mean arterial pressure were similar between Groups T and F, but increased in group C at 3 min after intubation (*p* < 0.001).

The incidences of tracheal carina mucosa injuries in all groups are shown in Table 3. The mucosal complication of tracheal carina was more frequently observed in group C. In the entrance of right main bronchus (region A), group C had the highest incidence of mucosa injury (19.4%, *p* = 0.03). In the entrance of left main bronchus (region B), the incidence of mucosa injury was lowest in Group T (8.3%, *p* < 0.01). In the left main bronchus (region C), the incidence of mucosa injury was lowest in Group T (25%, *p* < 0.001). In the main carina (region D), the incidence was highest in Group C (55.6%, *p* < 0.01). In the right main bronchus (region E), the incidence was also highest in Group C (44.4%, *p* < 0.001).

**Table 3:** Tracheal Carina Injury

	**Trachway® Group** **(T, *n* = 36)**	**Conventional Group** **(C, *n* = 36)**	***P* value** **(C VS T)**	**Fiberscope Group** **(F, *n* = 36)**	***P* value** **(F VS T)**
**Region A**	1/36(2.8%)	7/36(19.4%)	0.03	1/36(2.8%)	1
**Region B**	3/36(8.3%)	5/36(13.9%)	0.71	13/36(36.1%)	0.009
**Region C**	9/36(25%)	29/36(80.6%)	< 0.001	33/36(91.7%)	< 0.001
**Region D**	8/36(22.2%)	20/36(55.6%)	0.007	10/36(27.8%)	0.786
**Region E**	0/36(0%)	16/36(44.4%)	< 0.001	2/36(5.6%)	0.493

The incidences and severity of postoperative odynophagia was comparable between groups (Table 4). Most of the patients were categorized as none or mild pain only. The incidences of postoperative hoarseness and sore throat were not statistically significant among groups. The discomfort of postoperative hoarseness subsided gradually as days went by. By postoperative third day, no patient was in a stage of moderate or severe hoarseness. The severity of postoperative sore throat also decreased as days went by and only one patient in group C had moderately sore throat at second postoperative day.

**Table 4:** Postoperative odynophagia characteristics

	**Trachway Group (T, *n* = 36)**	**Conventional** **Group (C, *n* = 36)**	**Fiberscope Group (F, *n* = 36)**	***P* value**
**1**^**st**^** day**				
**Incidence**	9/36(25%)	15/36(41.67%)	15/36(41.67%)	0.236
**Severity** (none/mild/moderate/severe)	27/7/2/0	21/12/3/0	21/11/4/0	0.54
**2**^**nd**^** day**				
**Incidence**	3/36(8.33%)	2/36(5.56%)	6/36(16.67%)	0.246
**Severity** (none/mild/moderate/severe)	33/3/0/0	34/2/0/0	29/6/0/0	0.268
**3**^**rd**^** day**				
**Incidence**	3/36(8.33%)	2/36(5.56%)	2/36(5.56%)	0.858
**S** **everity** (none/mild/moderate/severe)	33/3/0/0	34/2/0/0	34/2/0/0	0.858
**4**^**th**^** day**				
**Incidence**	1/36(2.78%)	0/36(0%)	0/36(0%)	0.364
**Severity** (none/mild/moderate/severe)	35/1/0/0	36/0/0/0	36/0/0/0	0.364

## Discussion

In this study, our primary finding was that, compared with the other two groups, using the Trachway® flexible stylet for correct DLT placement took the shortest time. The mean DLT placement time for the conventional technique group was 146.8 s. Previous studies have reported that average time for conventional technique of placing DLT was 85 – 128 s [10–12]. However, in the study of Ruetzlar K *et al*. [10] and Schuepbach R *et al.* [12], the definition of time to initiate tube placement did not include time for bronchoscopy. In the study of Campos *et al.* [11], if the conventional technique failed to place the bronchial lumen in the left main bronchus at first attempt, they changed to use FFB immediately. In this study, there were three attempted chances to place the bronchial lumen correctly in all groups; therefore, it was reasonable to spend more time in Group C as compared with previous studies.

The success rates of correct placement in first attempt with the Trachway® flexible stylet (88.9%) and FFB (91.7%) were similar. However, the first success rate using the conventional technique was only 25% and much lower than previous reports [1, 13–15]. According to our observation, the modified bending technique of DLT we used [16] might have contributed to this unexpected result. Because of angling the bronchial lumen to conceal the tracheal lumen, we changed the natural curve of the left-sided DLT tip temporarily. This change might result in the bronchial lumen of left-sided DLT turning into the right main bronchus more easily. Besides, the metal stylet of DLT was withdrawn after the tracheal cuff of the DLT passed through the vocal cords and also increased the incidence of misplacement left-sided DLT into the right main bronchus [17]. The above two reasons might explain the low success rate of correct placement in group C. In Group T, although the metal stylet in the bronchus lumen was removed, the Trachway® flexible stylet in the tracheal lumen still maintained the rigidity and visibility during advancement of DLT. This could be the reason why the first success rate of Group T was similar to Group F.

Regarding the mucosa damage of the tracheal carina, we had enlisted the tracheal carina into five regions for observation. Group C had the highest incidence of mucosa injury in the entrance of the right main bronchus (region A) and the main carina (region D), and it was also higher than in other reports [12, 18]. The modified intubating technique we used should be the main reason for this. Since the incidence of misplacement left-sided DLT into the right main bronchus was highest in group C, the mucosa of the main carina and the right main bronchus were easily injured by the tip of the bronchial lumen. In Group F, under the guide of FFB, intubation of the bronchial lumen might be too deep and cause injuries in the entrance of left main bronchus (region B) and the left main bronchus (region C). In contrast, the DLT was able to synchronize the advancing tube at the same time in Group T. Mucosal injuries related to inadequate depth of the bronchial lumen could be prevented by direct vision of the Trachway® flexible stylet via the tracheal lumen. Moreover, the right main bronchus might be still mistaken for the trachea by FFB in Group F. Although it would be identified by auscultation of the stethoscope eventually, right main bronchus (region E) was injured in 2 out of 36 patients.

The postoperative side effects including sore throat, hoarseness, and odynophagia were comparable in all groups. In this study, all patients were intubated with the same brand of left-sided DLT, and all DLT intubations used the same technique [16]. Besides, duration of anesthesia longer than 2 h might be the risk factor of post-intubation sore throat [19], but this was also not different between all groups, so assuming no difference in post-intubation complications between all groups was reasonable.

Using flexible fiberoptic bronchoscope to confirm position of DLT is a standard procedure [20], but it is fragile and needs a high level of operating experience [21]. Although the Trachway® flexible stylet is unable to operate in direction, it is cheaper and more durable. The cost of maintaining and cleaning FFB might probably exceed USD $100 per case [22]. In this study, at least 36 cases used the Trachway® flexible stylet for correct placement of DLTs successfully, and this device has been used in our hospital for two years without any failure and damage. Accordingly, the Trachway® flexible stylet seems to be a cost-effective option for long-term use.

There are other airway devices that could reduce the use of FFB and increase successful rate of correct DLT placement. We believe that the Trachway® flexible stylet has at least two advantages. Firstly, the Trachway® flexible stylet could be inserted into the tracheal lumen of DLT easily in a commonly used size, such as 35F and 37F. If the camera tip were to be stained by sputum or discharge, it is easily retractable to clean and re-load without any movement of the DLT. Secondly, it revealed its advantage of real-time assessment of DLT placement to identify and correctly place bronchial lumen.

Although the primary outcome of our study is well-proven, some limitations exist. Firstly, it is rather difficult for anesthesiologists to execute intubation procedures without sufficient attention to the intubation tools. A large randomized study among medical centers should be launched to decrease any potential clinical bias. Secondly, different operation procedures and ease of operation were not evaluated in our study, although the anesthesiologists performing DLT placement were very familiar with these two airway devices. If the pulmonary disease progressed, inflammatory tissues might impede the bronchial lumen advancement and increase the incidence of side effects. Thirdly, a single brand left-side DLT was used in our study. Other DLTs such as right-sided DLTs or other brand tubes might change the results of the study.

In summary, comparing with conventional technique and FFB technique, the Trachway® flexible stylet for correct placement of left-sided DLT not only shortened the intubation time, but reduced carinal injuries as well. The conventional technique was associated with more mucosal lesions at the level of the carina and the right main bronchus. The Trachway® flexible stylet technique is worthy of application and is an option for good left-sided DLT placement.

## Supplementary Information

**Additional file 1.**

## Data Availability

All data generated or analysed during this study are included in this published article.

## Abbreviations

DLTs: Double-lumen endobronchial tubes
FFB: Flexible fiberoptic bronchoscope
OLV: One-lung ventilation
